# Prospective study on health-related quality of life, oral mucositis and oral health on during treatment of head and neck cancer

**DOI:** 10.1186/s12903-024-04466-5

**Published:** 2024-06-15

**Authors:** Charlott Karlsson, Niklas Bohm, Jessica Skoogh Andersson, Caterina Finizia, Annica Almståhl

**Affiliations:** 1 Section 4- Oral Health, Malmö University, Malmö, Sweden; 2 Department of Oral and Maxillofacial Surgery, Institute for Postgraduate Dental Education, Jönköping, Sweden; 3 Deptartment of Oral Microbiology and Immunology, Institute of Odontology, Sahlgrenska Academy, University of Gothenburg, Göteborg, Sweden; 4 Deptartment of Periodontology, Institute of Odontology, Sahlgrenska Academy, University of Gothenburg, Göteborg, Sweden; 5 Department of Otorhinolaryngology, Head and Neck Surgery, Institute of Clinical Sciences, Sahlgrenska Academy, University of Gothenburg, Göteborg, Sweden; 6 Department of Otorhinolaryngology, Head and Neck Surgery, Region Västra Götaland, Sahlgrenska University Hospital, Gothenburg, Sweden

**Keywords:** Head and neck cancer, Oral mucositis, Health related quality of life, Radiotherapy, Saliva, Oral hygiene

## Abstract

**Background:**

Few studies have examined health related Quality of Life (HR-QoL) during the treatment of head and neck cancer (HNC) with even fewer focusing on the impact of oral mucositis (OM) on HR-QoL. Studies performed during treatment of HNC makes it possible to follow fluctuations in HR-QoL, OM and other treatment related side effects. The aim was to prospectively analyze HR-QoL, changes in clinical variables and the impact of OM on HR-QoL during HNC treatment.

**Materials and methods:**

Patients were recruited before commencing curative cancer treatment and were given professional oral care weekly during oncologic treatment. HR-QoL was reported before, during (week 2, 4 and 6) and three months after treatment using the EORTC Quality of Life questionnaires C30 and H&N35 and the stimulated whole salivary secretion rate was determined at the same time-points. OM (erythema and ulceration) was registered using the Oral Mucositis Assessment Scale (OMAS), at baseline, weekly during treatment and post treatment. Differences in HR-QoL between different timepoints were analyzed. To analyze the impact of OM on HR-QoL the patients were categorized into two groups: no/mild OM (OMAS ulceration score 0–1) or severe OM (OMAS ulceration score ≥ 2) and HR-QoL was compared between the two OM groups at three timepoints during treatment.

**Results:**

Fifty-seven patients (43 men, 14 women), with a mean age of 58 years were included. Patients reported progressively impaired HR-QoL, with peak issues noted at weeks 4 and 6, particularly in social eating, senses, appetite loss, sticky saliva, and decreasing salivary secretion rates were determined. Patients with severe OM reported worse HR-QoL compared to those with no/mild OM. Persistent problems 3 months post treatment were appetite loss, dry mouth, senses (smell and taste) and problems with social eating.

**Conclusion:**

Patients experienced exacerbated symptoms and problems weeks 4 and 6 of oncological treatment, especially among those with severe OM, stressing the importance of clinically monitoring the patients to reduce and alleviate their symptoms. Persistent problems three months post treatment are likely associated with the reduced salivary secretion rate indicating that patients should be monitored also after completed oncological treatment.

## Introduction

Head- and neck cancer (HNC) encompasses tumors in the lips, oral cavity, oropharynx, nasopharynx, nose and sinuses, larynx, major salivary glands, and head- neck cancers of unknown primary tumors (HNCUP) [1]. The anatomical and functional complexity of the head and neck area significantly influences overall appearance and functioning [2].

HNC ranks as the seventh most common type of cancer worldwide, with approximately 150,000 new cases reported annually in Europe [3]. In Sweden there are around 1600 new cases reported each year [4]. Lifestyle factors primarily contribute to the risk of HNC, with smoking being the strongest independent risk factor. Combining smoking with alcohol consumption further increases the risk [5]. Additionally, low intake of fruits and vegetables increases susceptibility to HNC [6]. It has also been shown that poor dental status and poor oral hygiene are other independent risk factors for oral and oropharyngeal HNC irrespective of tobacco and alcohol consumption [7]. Chronic mechanical irritation from poorly fitting dentures, rubbing against the gingiva or mucous membrane, could be a risk factor for HNC [8]. Human papilloma virus (HPV) is increasingly associated with HNC, particularly in the oropharynx, a globally observed trend including in Sweden [9].

Curative treatment for HNC tumors involves various combinations of radiotherapy (RT) chemotherapy and surgery [4]. These oncological treatments impact essential functions such as breathing, chewing, swallowing, the senses (taste and smell) and speaking [10]. Common symptoms related to treatment include pain, oral dryness, fatigue, nutritional problems, weight loss [11], and restricted mouth opening (trismus) [12], all of which negatively affect health-related quality of life (HR-QoL).

HR-QoL encompasses the subjective perception of various cancer- related aspects, including physical, emotional, social and cognitive functions, along with disease symptoms and side effects [13]. Numerous studies have prospectively measured HR-QoL before, during and shortly after oncologic HNC treatment [14–22]. Patients in these studies report diminished HR-QoL over time during treatment regarding fatigue, nausea and vomiting, dry mouth, sticky saliva, swallowing, sensory changes, and symptomatic dental problems. However, none of these studies evaluating HR-QoL [14–22] provided information on clinical dental status, dental treatment, and supportive oral care before and during oncologic HNC treatment.

Oral mucositis (OM), an acute injury and inflammation of the oral mucosa resulting from HNC treatment, may contribute to several of these symptoms [23, 24]. The incidence of OM in HNC exceeds 85% with around 66% estimated to have severe OM (ulcerations). OM often begins as erythema with subsequent erosion and ulceration, often covered by a white fibrinous pseudomembrane primarily affecting non-keratinized oral mucosa (lateral and ventral tongue, buccal mucosa and soft palate). These lesions weaken the barrier of the mucosa potentially leading to local or systemic infections [24].

The incidence and severity of OM depend on factors such as tumor site and type or intensity of treatment, where combined RT and chemotherapy could potentially exacerbate the severity and duration of OM [24]. Patient related factors such as gender, low performance status in daily living abilities at baseline and comorbidities may influence severity of OM [25]. OM is associated with pain, difficulty in oral hygiene maintenance, dysphagia, talking, eating and drinking difficulties and weight loss [24]. To the best of our knowledge, only two studies have evaluated both HR-QoL and OM prospectively before and during HNC treatment [16, 20], revealing a statistically significant detrimental effect of OM particularly regarding pain. However, even though poor dental status and oral hygiene are suggested to increase the risk of OM and worsen symptoms of OM, information regarding dental treatment and supportive oral care was lacking in these studies [16, 20].

The aim of this study was to prospectively analyze HR-QoL, changes in clinical variables and the impact of OM on HR-QoL during HNC treatment.

## Materials and methods

This study is part of a broader project aimed at evaluating an oral care protocol for patients undergoing treatment for HNC. Patients were consecutively recruited from four regions in Sweden prior to starting oncologic treatment. Inclusion criteria comprised individuals aged ≥ 18 years with ≥ 16 own teeth, scheduled to receive curative oncologic treatment including full dose RT. The exclusion criteria included recurrent cancer and/or severe cognitive impairment (such as dementia, brain injury or disabilities hindering comprehension of written text). Patients meeting the inclusion criteria received oral- and written information about the study and informed consent was obtained.

### Oral management

In Sweden, patients scheduled for HNC treatment including RT undergo a thorough oral/dental examination including X-rays to assess oral health and prevent dental infections during and after oncologic treatment. Teeth with extensive periapical disease, moderate to severe attachment loss or severe decay teeth are extracted, and manifest caries lesions restored prior to the start of oncologic treatment. An assessment of oral hygiene is conducted with individual oral hygiene instructions provided. Conventional periodontal therapy is administered if necessary.

### Oral care protocol in the study

Patients visited a dental hygienist before commencing oncologic treatment and received advice on prescribed practices during oncologic treatment: (1) brushing teeth twice daily with 2 cm fluoride toothpaste (1450 ppm) for at least 2 min, (2) cleaning interdentally once daily, rinsing with 0.2% sodium fluoride daily, (3) abstaining from tobacco and alcohol. Local pain relief was prescribed according to the patient’s needs. Professional oral care was administered by a dental hygienist once weekly until symptoms of OM subsided.

### Oncologic treatment

All patients underwent salivary gland sparing radiotherapy (RT) with Intensity-Modulated Radiation Therapy IMRT or Volumetric Modulated Arc Therapy (VMAT), at a rate of 2 Gy/day, 5 days per week over 5–7 weeks, resulting in a total dose of 68–70 Gy. Some of the patients who underwent RT received Cisplatin based chemotherapy weekly during RT, so called chemoradiotherapy (CRT). Patients who underwent surgery also received RT or CRT.

### Data from medical files/ medical records

Patient medical records were reviewed to gather information on height, weight, comorbidities, and medications. Additionally, details regarding HNC diagnosis, tumor site, radiation dosage and medical tumor treatment were extracted. Body Mass Index (BMI) was calculated accordingly.

### Clinical examination

Throughout all assessments, the patients were asked regarding their smoking habits and alcohol consumption. A clinical examination including registration of number of teeth, restored teeth, plaque and gingival inflammation was conducted prior to the start of oncologic treatment (baseline) and at three months post-treatment. Plaque and gingival inflammation were also assessed during weeks 2, 4 and 6 of RT. Plaque and gingival inflammation were registered visually and/or with a periodontal probe on the six Ramfjord teeth. Plaque was graded from 0 to 3 according to Silness and Löe [26], while gingival inflammation was graded from 0 to 3 according to Löe and Silness [27].

### Stimulated whole salivary secretion rate

Salivary secretion rate was measured at baseline, weeks 2, 4 and 6 during treatment and three months post oncologic treatment. Patients were instructed to abstain from eating, drinking (except water), tooth-brushing, and smoking one hour prior to their appointment. The stimulated whole salivary secretion rate was measured using paraffin wax. Patients were asked to chew on a piece of paraffin until softened and then to swallow the saliva produced once. Subsequently patients chewed on the paraffin at their own pace and spat out all saliva continuously in a test tube over a period of five minutes.

### Maximum interincisal opening

Maximal interincisal opening (MIO) was assessed before treatment, at week 6 of RT and at one and three months post oncologic treatment. MIO was measured with a ruler in an upright position as the maximum distance between the upper and lower incisors. Values ≤ 35 mm as defined by Dijkstra et al. [28], indicated problems with mouth opening.

### EORTC QLQ C30 and EORTC H&N 35

The European Organization for Research and Treatment of Cancer (EORTC) Quality of life Questionnaire Core 30 (QLQ C30) is a Patient Reported Outcome (PRO) questionnaire used to evaluate HRQL in cancer patients [29]. Comprising 30 questions, it includes five functioning domains, a global quality of life scale, three symptom domains and six single items describing patients’ symptoms and functional level over the past week. The EORTC quality of life questionnaire head and neck module (H&N-35) is a diagnose specific questionnaire designed HNC patients [30]. Consisting of 35 items condensed into seven multi-item symptom domains and 11 single items, it covers various aspects including pain, swallowing, senses (taste and smell), speech, social eating, social contact, and sexuality, as well as problems related to teeth, mouth opening, oral dryness, sticky saliva, coughing, feeling ill, weight, and nutrition. Both questionnaires result in scales/domain scores with functioning domains and global quality of life (QOL) calculated on a scale from 0 to 100 (where 100 represents maximum functioning and global quality of life), while symptom domain and single items, are scored inversely with 100 indicating worst possible symptoms and problems. A change in score of > 10 points between time-points is considered clinically significant [31].

### Oral mucositis

OM was registered with the patient sitting or lying in a dental treatment chair with the oral cavity illuminated by medical grade operating light with use of an oral mirror if needed. The clinician reported Oral Mucositis Assessment Scale (OMAS) [32] was used, registering OM across nine intraoral sites: (1) upper lip, (2) lower lip, (3) left side of the buccal mucosa, (4) right side of the buccal mucosa, (5) left ventral and dorsal side of the tongue, (6) right ventral and dorsal side of the tongue, (7) floor of the mouth, (8) soft palate and (9) hard palate. Ulceration was scored from 0 to 3, (0 = no ulceration, 1 = < 1 cm^2^, 2 = 1–3 cm^2^, 3 = > 3 cm^2^) and erythema from 0 to 2 (0 = no erythema, 1 = mild erythema, 2 = severe erythema) resulting in total ulceration scores ranging from 0 to 27 and erythema scores ranging from 0 to 18. OM was evaluated at baseline, weekly during oncologic treatment, and at one- and three months post treatment. All measurements were conducted by six experienced dental hygienists who underwent training together via workshops to reach consensus regarding the assessment of clinical variables.

### Statistical methods

All data was checked for normal distribution, and it was shown that they were not normally distributed why non-parametric tests were used. Mean values and standard deviations (SD) were calculated for the number of teeth, plaque, gingival bleeding, stimulated salivary secretion rate and weight and Sign test was used to analyze differences between baseline and 3 months post treatment. For HR-QoL data, mean values and 95% confidence intervals (CI) were calculated. Differences in scores exceeding 10 points between timepoints were deemed clinically significant [31]. Statistically significant differences in HR-QoL between time-points (baseline - week 2, week 2 - week 4, week 4 - week 6, week 6 − 3 months post treatment, and between baseline and three months post treatment were analyzed using Wilcoxon signed-rank test. According to OMAS ulceration scores, the patients were categorized into two groups: no/mild OM group (ulceration scores 0–1 points), and severe OM group (ulceration scores ≥ 2 points). Differences in HR-QoL scores between the two OM groups were evaluated at week 2, 4 and 6 during treatment using the Mann-Whitney U-test. P-values of < 0.05 were considered statistically significant.

### Ethical considerations

This study adhered the principles outlined in the Declaration of Helsinki. Approval was obtained by the Regional Ethics Committee at the University of Gothenburg Dnr 831 − 16.

## Results

### Patients

A total of 57 patients were enrolled between 2018 and 2022. The mean age was 58 years (range 22–79 years), with 43 (75%) being males. Normal BMI was observed for 34% of the patients at baseline and in 47% at the three months follow-up (Table 1). Weight loss progressed gradually during oncologic treatment with 34% losing more than 10 kg to 23 kg, and 32% losing between 5 and 10 kg by three months post treatment. The mean weight significantly decreased to 79 ± 13 kg by three months post treatment, compared to baseline, 85 ± 14 kg (*p* < 0.001). 63% of the patients were never smokers (Table 1). Compared to baseline (Table 1), the percentage of patients reporting alcohol consumption had decreased to 17% by week 2 of treatment. The corresponding figures for weeks 4 and 6 were 8% and 13%, respectively.

**Table 1:** Body mass index, smoking status, alcohol use, medicines and diseases/conditions for the 57 patients at baseline. Tumor location and treatment are also presented

BMI	
Under weight (no/%)	2 (4)
Normal weight	17 (34)
Over weight	21 (42)
Obese	10 (20)
Smoking status	
Current smoker	3 (5)
Former smoker quitted < 3 months ago	4 (7)
Former smoker quitted years ago	14 (25)
Never smoked	36 (63)
Alcohol use	
None	19 (33)
Liquor	4 (7)
Other (wine, beer)	29 (51)
Both liquor and other	5 (9)
Diseases/conditions	
None	37 (65)
1	8 (14)
2-4	12 (21)
Medicines	
None	17 (30)
1–3	17 (30)
4–8	23 (40)
Tumor location	
Oropharynx	40 (70)
Oral	5 (5)
Larynx	5 (5)
Salivary glands	3 (5)
Nasopharynx	2 (3)
HNCUP	2 (3)
Treatment	
Chemo+RT	34 (60)
RT	16 (28)
S+RT	6 (11)
S+ Chemo+RT	1 (1)

Chemo = chemotherapy, RT = radiation therapy, S = surgery

HNCUP = head- neck cancer of unknown primary tumor

Additionally, 65% of the patients (*n* = 37) had no comorbidities except for HNC (Table 1). Among the 20 patients with comorbidity, hypertension (*n* = 8), heart failure (*n* = 5), and diabetes mellitus type 2 (*n* = 4) were the most common. At baseline 40 patients (70%) were using medication (Table 1), with anti-hypertensives (*n* = 25, 64%), painkillers (*n* = 24, 60%), statins (*n* = 4, 7%) and proton pump inhibitors (*n* = 4, 7%) being the most common.

The majority of the patients were diagnosed with oropharynx cancer (*n* = 40, 70%), with CRT being the most common oncologic treatment (*n* = 34, 60%). Among patients with oropharynx cancer (*n* = 40), 28 underwent CRT while 12 received RT. Among those with oral cancer (*n* = 5), three underwent surgery and RT, one received CRT and one underwent RT only. One patient with spread gingival cancer had a surgical removal of a part of the mandibular jawbone (mandibulectomy) and half of the tongue (hemi glossectomy) followed by RT. Among patients with larynx cancer (*n* = 5), three received CRT, one underwent RT, and one underwent surgery followed by CRT. Among patients with salivary glands cancer (*n* = 3), two underwent surgery and RT and one received RT only. Two patients had nasopharynx cancer with one undergoing RT and the other undergoing maxillectomy followed by RT. Among patients with HNCUP (*n* = 2), one received RT and one received CRT.

### Dental status

The mean number of teeth at baseline was 27 (range 19–32) (Table 2). The mean plaque score was low at baseline 0.53 ± 0.60, decreasing further to 0.35 ± 0.38 at three months post treatment (*p* < 0.01). Similar results were observed for gingivalinflammation, with mean scores of 0.43 ± 0.51 at baseline and 0.26 ± 0.34 at three months post treatment (*p* < 0.01) (Table 2).

**Table 2:** Number of teeth, plaque, gingival bleeding, mouth opening and stimulated salivary secretion rate (ml/min) at baseline and 3 months post treatment for the 57 patients

	Baseline	3 months post RT	*P*-values
Number of teeth			
Mean ± SD	27±3.5	27±3.6^a^	N.S
Median	28	28	
Range	19-32	18-32	
Plaque^b^			
Mean ± SD	0.53±0.60	0.35±0.38	*p* < 0.01
Median	0.33	0.25	
Range	0.00-2.50	0.00-1.88	
Gingival bleeding^c^			
Mean ± SD	0.43±0.51	0.26±0.34	*p* < 0.01
Median	0.21	0.13	
Range	0.00-2.00	0.00-1.50	
Mouth opening MIO ≤35 mm	4 patients (7%)	5 patients (9%)	
Stimulated salivary secretion rate (ml/min)d			
Mean ± SD	1.7±0.7	0.7±0.5	*p* < 0.001
Median	1.7	0.6	
Range	0.4-4.0	0.0-2.0	
Hyposalivation (<0.7 ml/min) No (%)			
	3 (5.3)	29 (56.9)	
Low secretion rate ≥ 0.7-1.0 ml/min No (%)			
	2 (3.5)	12 (23.5)	
Normal (> 1.0 ml/min) No (%)			
	52 (91.2)	10 (19.6)	

a: data missing for 4 patients

b: data missing for 1 patient at baseline and for 5 patients 3 months post treatment

c: data missing for 2 patients at baseline and for 5 patients 3 months post treatment

d = salivary secretion rate could be determined in 56 patients at baseline and in 50 patients 3 months post oncological treatment

### Mouth opening

Four patients had a reduced capability of mouth opening (MIO 25–33 mm) at baseline (Table 2). The number of patients with MIO ≤ 35 mm increased week 6 of oncologic treatment (*n* = 15, 26%). Fifteen patients (26%) had MIO ≤ 35 mm at a single time- point. At three months post treatment, five patients (9%) had reduced mouth opening capability (MIO 23–30 mm). The patients with MIO < 35 mm at baseline exhibited a decreased MIO at all time-points.

### Salivary secretion rate

The mean salivary secretion rate at baseline was 1.7 ml/min with 91% of patients having a normal secretion rate of > 1.0 ml/min. The mean reduction in salivary secretion rate was 18% by week 2 of RT in 44 patients where measuring of salivary secretion rate could be determined and 38% by week 4 of RT in 44 patients where the secretion rate was determined. At three months post treatment, the mean secretion rate was 0.7 ml/min with hyposalivation (< 0.7 ml/min) observed in 40% of patients (*n* = 23) and low secretion rates observed in 21% (*n* = 12) (≥ 0.7-1 ml/min) (Table 2). The mean reduction in salivary secretion rate compared to baseline was 54% (median 63%).

### Oral mucositis

**Fig. 1:**
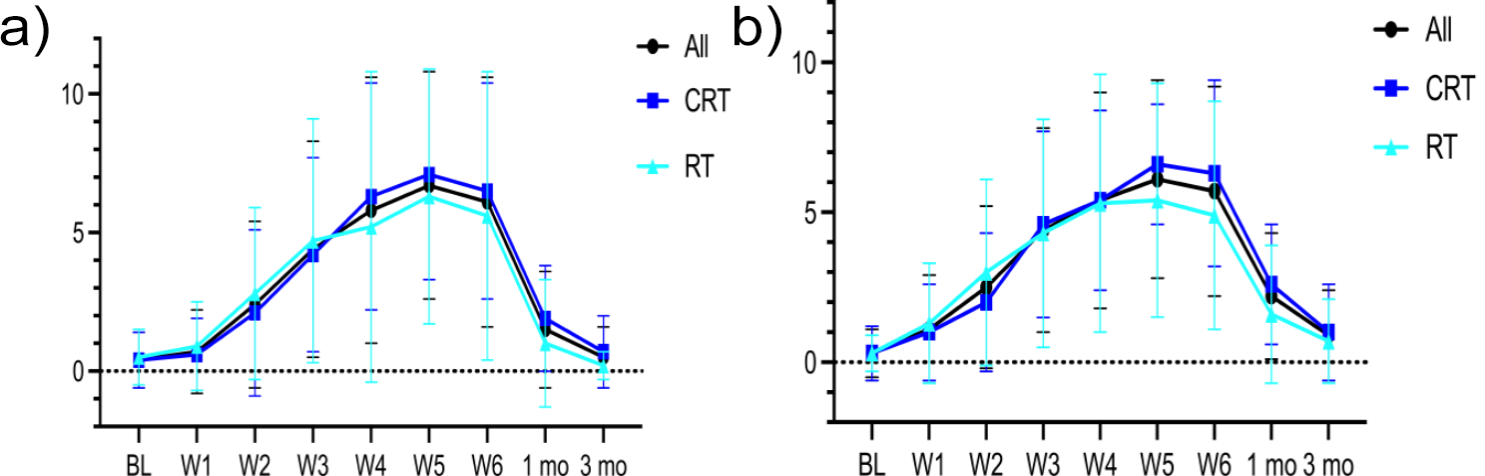
**a**. Ulceration scores (Mean ± SD) at the different time-points for all patients (*n* = 57) and for the patients who were treated with CRT (*n* = 24) and the patients who were treated with RT (*n* = 33), respectively. **b**. Erythema scores (Mean ± SD) at the different time-points for all patients (*n* = 57) and for the patients who were treated with CRT (*n* = 24) and the patients who were treated with RT (*n* = 33)

The highest mean ± SD scores for ulceration and erythema were recorded at week five of oncologic treatment (6.7 ± 4.1 and 6.1 ± 3.3, respectively) (Fig. 1a and b). Both ulceration- and erythema scores varied markedly among the 57 patients at the different time-points (Fig. 1a and b), with the worst scores consistently recorded at weeks 4–6 during oncologic treatment. Patients treated with CRT exhibited slightly higher mean scores for both ulceration (Fig. 1a) and erythema (Fig. 1b) at weeks 5 and 6 of oncologic treatment, compared to those treated with RT.

**Fig. 2:**
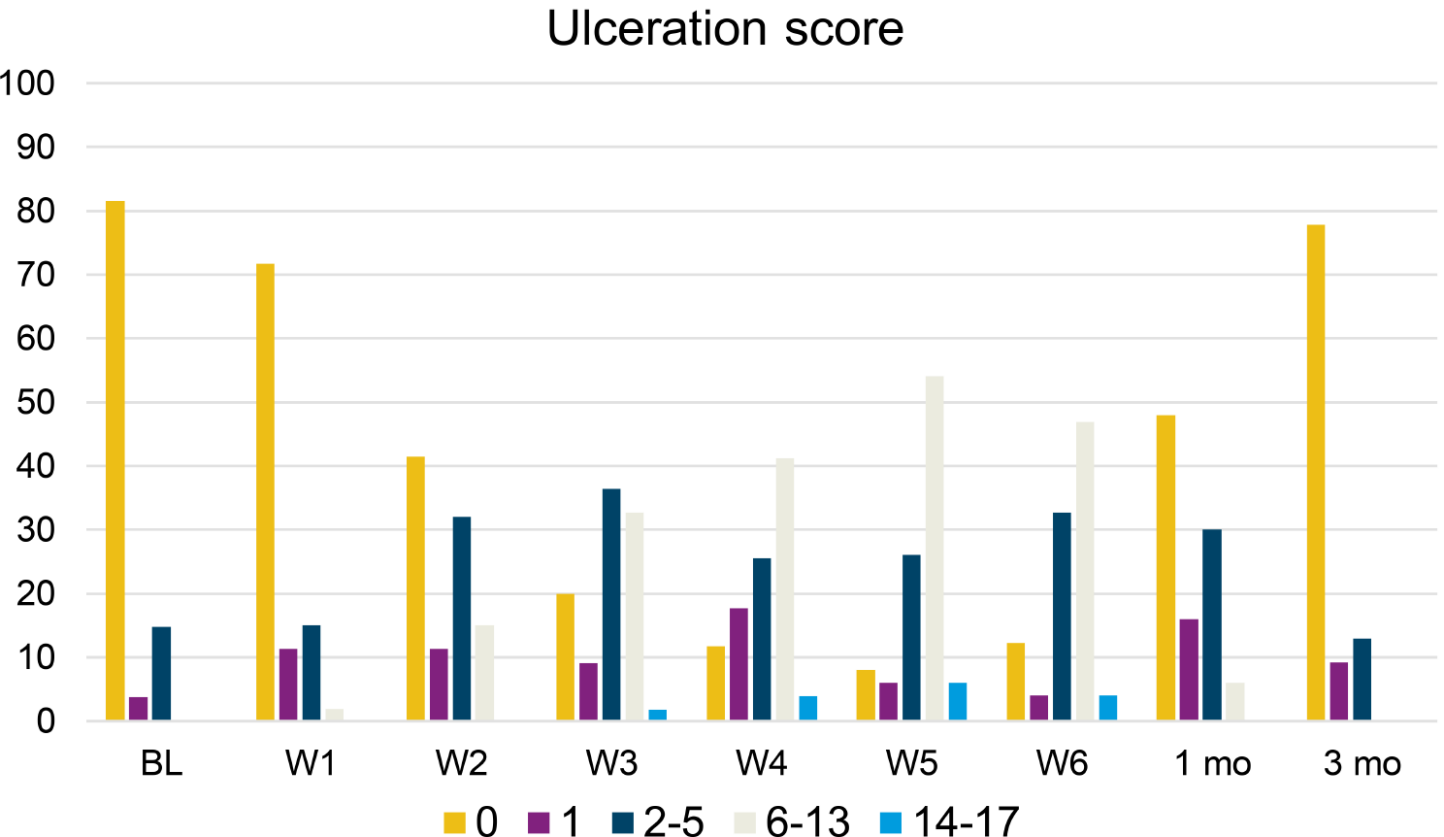
Proportion of patients with ulceration scores of 0, 1, 2–5, 6–13 and 14–17 at baseline, weekly during oncological treatment and 1- and 3-months post treatment. Missing values: BL *n* = 3, W1 *n* = 4, W2 *n* = 4, W3 *n* = 2, W4 *n* = 6, W5 *n* = 7, W6 *n* = 8, 1 mo. *n* = 7, 3 mo.*n* = 3

An increasing proportion of patients showed ulcerations and increased scores each week of treatment, with the ulceration score peaking at week 5 of treatment with 60% showing scores between 6 and 17 (Fig. 2).

### EORTC QLQ-C30

The first symptoms reported by the patients were appetite loss and constipation at week 2 of treatment (Table 3). By week 4 of treatment, clinically and statistically significant impaired functioning was reported across most domains particularly notable in role functioning (*p* < 0.001). Patients also reported a decrease in global QoL (*p* < 0.001) as well as for three of the symptom domains and single items, with the largest increases seen for appetite loss (*p* < 0.001) and pain (*p* < 0.001) (Table 3). At week 6 of RT, most scores remained similar to those reported at week 4.

**Table 3:**
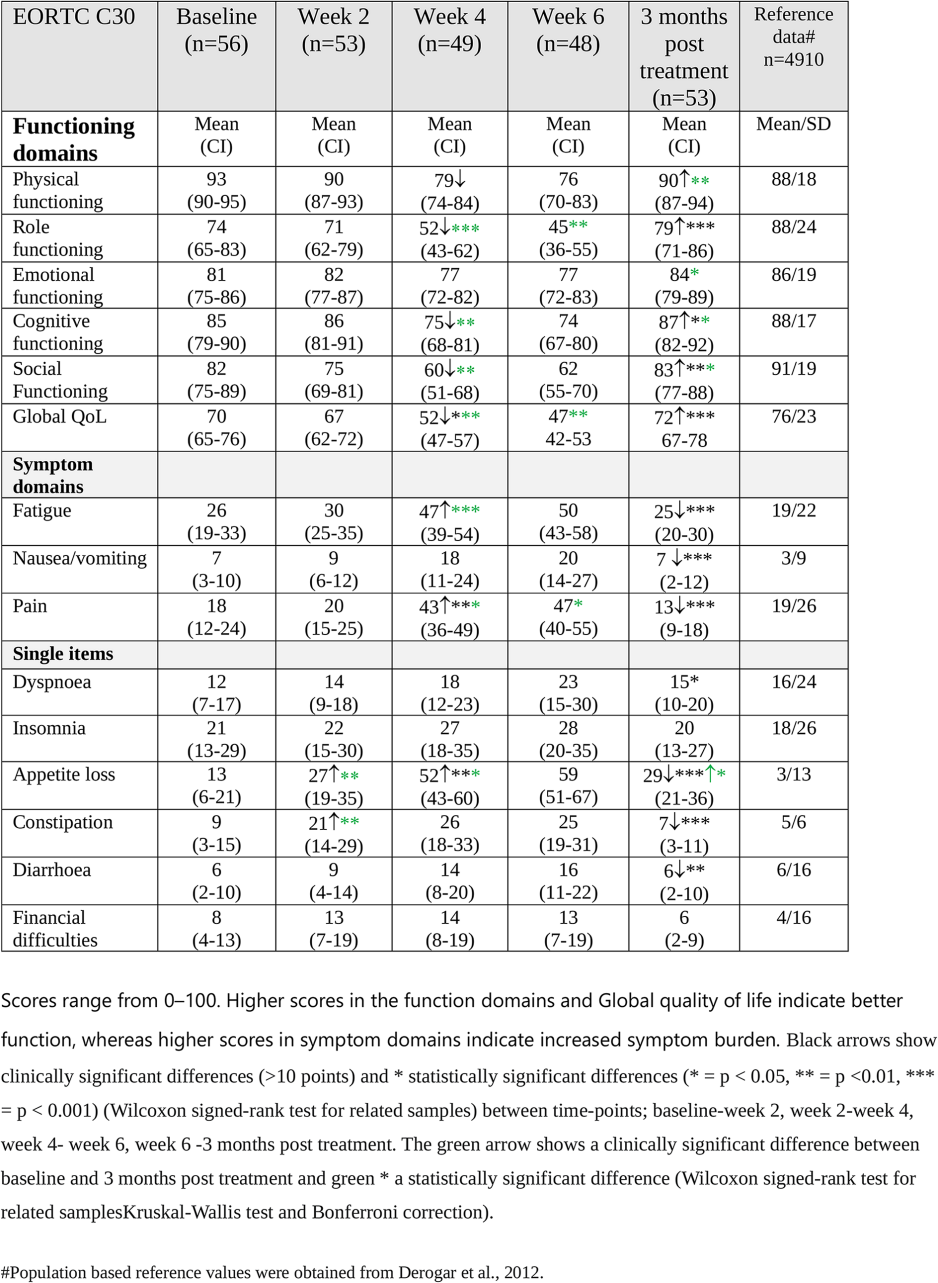
EORTC QLQ C30 scores before, during and three months post treatment for the 57 HNC patients. Mean values and 95% confidence intervals (CI) are presented

At three months post treatment, patients reported clinically and statistically significant improvement for 10 of the functioning/symptom domains and single items (Table 3). The most substantial improvements were observed for pain (*p* < 0.001), role functioning (*p* < 0.001), appetite loss (*p* < 0.001), fatigue (*p* < 0.001) and global QoL (*p* < 0.001) (Table 3). Compared with baseline, patients reported increased problems only with appetite loss at three months post treatment (*p* < 0.05). Compared to population-based reference values [33], patients scored worse on 13 of the 15 functioning/symptom domains and single items at least at one time-point (Table 3), and clinically significantly more problems with role functioning and appetite loss at all time-points during treatment.

### EORTC QLQ H&N 35

By week 2 of treatment, the patients reported more problems in five symptom domains and single items with the most notable increases seen for sticky saliva (*p* < 0.001), dry mouth, (*p* < 0.001) and senses (*p* < 0.001) (Table 4). At week 4 of treatment, the patients reported clinically and statistically significantly more symptoms and problems on all domains/scales and single items, except problems with teeth. Worst symptoms/problems were reported for social eating (*p* < 0.001), senses (*p* < 0.001), and swallowing (*p* < 0.001). There was also a marked increase in the use of painkillers, need for nutritional support, and problems with weight loss (Table 4). Worsening problems and symptoms were reported at week 6 of treatment (Table 4).

**Table 4:**
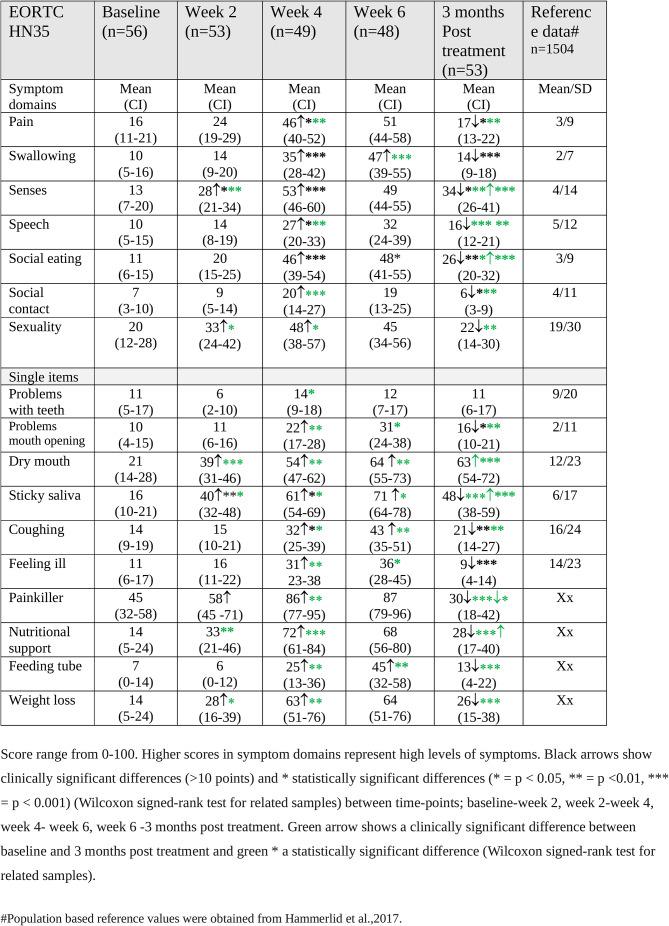
EORTC QLQ H&N 35 scores before, during and 3 months post treatment for HNC. Mean values and 95% confidence intervals (CI) are presented

At three months post treatment, the patients reported reduced symptoms and problems for 15 of the 17 symptom domains/single items. The largest decreases were seen for head and neck pain (*p* < 0.05) as well as swallowing problems (*p* < 0.001) and for the single items, feeling ill (*p* < 0.001) and sticky saliva (*p* < 0.001). However, problems with dry mouth remained high (Table 4). Compared to baseline, the patients reported clinically and statistically significantly more problems with especially dry mouth (*p* < 0.001), sticky saliva (*p* < 0.001, senses (*p* < 0.001) and social eating (*p* < 0.001) at three months post treatment. In comparison with population-based reference values [34], the patients reported more problems on 13 of the 14 symptom domains/single items at least at two time-points, with the most substantial differences observed for sticky saliva and head and neck pain.

### EORTC C30 and OM

From baseline to week 2 of treatment patients with severe OM reported clinically significantly more problems with constipation compared to patients with no/mild OM (Table 5). By week 4 of treatment, the patients with severe OM reported clinically and/or statistically significantly more problems for 14 of the 15 functioning domains, symptom domains and single items, with notable differences observed in role functioning (*p* < 0.001), social functioning (*p* < 0.001) as well as pain (*p* < 0.001) and fatigue (*p* < 0.05) (Table 5). At week 6, the patients with severe OM reported clinically significantly more problems for 7 out of 15 functioning/symptom domains and single items with the greatest differences seen in social functioning, pain (*p* < 0.05) and insomnia. At three months post treatment, the patients with severe OM at week 6 of treatment reported clinically significantly more problems with appetite loss (Table 5).

**Table 5:** EORTC QLQ C30 scores before, during and post treatment. Scores at 3 months post treatment for those who had no/mild OM and severe OM week 6 of RT are also shown. Mean values and 95% confidence intervals (CI) are presented

EORTC C30	Week 2 No/mild *n* = 26	Week 2 Severe *n* = 25	Week 4 No/mild *n* = 14	Week 4 Severe *n* = 34	Week 6 No/mild *n* = 6	Week 6 Severe *n* = 40	3 mo No/mild at Week 6 *n* = 8	3 mo Severe At Week 6 *n* = 47
Functioning domains	Mean (CI)	Mean (CI)	Mean (CI)	Mean (CI)	Mean (CI)	Mean (CI)	Mean (CI)	Mean (CI)
Physical functioning	89 (86–93)	90 (85–95)	90 (81–98)	75$\downarrow$* (68–82)	84 (69–100)	77 (71–84)	93 (85–102)	90 (86–93)
Role functioning	68 (55–81)	72 (60–84)	75 (57–93)	42$\downarrow$** (31–52)	64 (33–95)	43$\downarrow$ (32–54)	83 (65–102)	78 (70–86)
Emotional functioning	82 (75–89)	82 (74–90)	85 (76–95)	74$\downarrow$* (68–81)	83 (69–97)	78 (71–84)	86 (75–98)	83 (78–89)
Cognitive functioning	86 79–93	87 (78–96)	88 (79–97)	69$\downarrow$* (60–78)	89 (78–100)	72$\downarrow$ (64–80)	92 (79–104)	86 (80–92)
Social Functioning	74 (64–85)	75 (67–83)	81 (66–95)	51$\downarrow$** (40–62)	83 (63–104)	61$\downarrow$ (52–70)	88 (74–101)	82 (75–88)
Global QoL	69 (62–76)	65 (58–72)	61 (48–74)	48$\downarrow$ (42–54)	56 (31–80)	46$\downarrow$ (40–52)	71 (55–87)	73 (67–79)
**Symptom domains**								
Fatigue	27 (20–35)	34 (26–41)	30 (16–44)	53$\uparrow$* (44–63)	44 (19–70)	50 (41–59)	24 (10–37)	26 (20–31)
Nausea/vomiting	6 (2–11)	12 (6–18)	12 (2–22)	21$\uparrow$ (12–29)	17 (0–34)	21 (13–29)	8 (2–15)	7 (1–12)
Pain	21 (13–28)	21 (14–28)	26 (15–37)	50$\uparrow$** (41–58)	22 (15–29)	50$\uparrow$* (41–58)	10 (0–21)	14 (8–19)
**Single items**								
Dyspnea	15 (8–23)	11 (4–17)	12 (3–21)	21$\uparrow$ (13–28)	22 (-5-50)	21 (13–29)	13 (1–24)	16 (10–21)
Insomnia	21 (10–31)	25 (13–38)	19 (6–32)	30$\uparrow$ (19–42)	17 (2–31)	30$\uparrow$ (20–40)	25 (9–41)	19 (12–27)
Appetite loss	23 (12–34)	32 (20–44)	45 (26–64)	55$\uparrow$ (44–66)	56 (34–77)	58 (48–68)	17 (4–29)	30$\uparrow$ (22–39)
Constipation	17 (7–26)	27$\uparrow$ (14–40)	21 (5–38)	28 (19–38)	22 (0–44)	26 (18–33)	4 (-4-12)	7 (3–12)
Diarrhoea	10 (2–19)	8 (1–15)	7 (0–15)	17$\uparrow$ (9–25)	6 (-5-16)	18$\uparrow$ (10–25)	13 (-5-30)	4 (0–8)
Financial difficulties	17 (6–28)	10 (3–17)	5 (-2-11)	18$\uparrow$ (9–26)	6 (-5-16)	14 (7–22)	4 (-4-12)	6 (2–10)

Week 2: Data missing for 2 patients in the no/mild group and for 4 patients in the severe group (*n* = 57)

Week 4: Data missing for 3 patients in the no/mild group and for 6 patients in the severe group (*n* = 57)

Week 6: Data missing for 2 patient in the no/mild group and for 1 in the severe group. OMAS data is missing for 8 patients (*n* = 57)

3 months: Data missing for 2 patients in the severe group (*n* = 57)

High scores for functioning domains represents high level of functioning and high scores for symptom domains represent high levels of symptoms. The arrows show clinicicallly significant differences (> 10 points) between the patients with no/mild OM and those with severe oral mucositis. * = *p* < 0.05, ** = *p* < 0.01 (Mann Whitney U test)

### EORTC H&N 35 and OM

Already by week 2 of treatment, the patients with severe OM reported clinically and/or statistically significantly more problems/symptoms with head and neck pain (*p* < 0.05), use of painkillers (*p* < 0.05), dry mouth, weight loss, nutritional support and the need for a feeding tube compared with those with no/mild OM (Table 6). Clinically and/or statistically significantly increased problems for 13 of the 17 symptom domains and single items were reported by the patients with severe OM at week 4 of treatment compared with patients with no/mild OM. The largest differences were seen for sticky saliva (*p* < 0.01) and problems with speaking (*p* < 0.01) (Table 6). At week 6 of treatment, patients with severe OM reported clinically and/or statistically significantly more problems particularly with mouth opening (*p* < 0.05), pain (*p* < 0.05) and swallowing (Table 6).

Three months post treatment, patients who had severe OM at week 6 of treatment reported clinically significantly more problems for sticky saliva, use of painkillers, the need for a feeding tube and weight loss compared to those who had no/mild OM at week 6 of treatment.

**Table 6:** EORTC QLQ H&N35 scores week 2, 4 and 6 of RT for the patients with no/mild OM and the patients with severe OM. Scores at 3 months post treatment for patients with severe OM week 6 of treatment are also shown. Mean values and 95% confidence intervals (CI) are presented

EORTC HN35	Week 2 No/mild *n* = 26	Week 2 Severe *n* = 25	Week 4 No/mild *n* = 14	Week 4 Severe *n* = 34	Week 6 No/mild *n* = 6	Week 6 Severe *n* = 40	3 mo No/mild at Week 6 *n* = 8	3 mo Severe at Week 6 *n* = 47
**Symptom** domains	Mean (CI)	Mean (CI)	Mean (CI)	Mean (CI)	Mean (CI)	Mean (CI)	Mean (CI)	Mean (CI)
Pain	18* (10–25)	30$\uparrow$* (23–37)	34 (24–43)	51$\uparrow$* (43–59)	28 (16–39)	54$\uparrow$* (46–62)	13 (4–22)	18 (13–23)
Swallowing	11 (3–18)	18 (8–28)	20 (8–33)	42$\uparrow$* (32–51)	24 (-1-48)	50$\uparrow$ (41–59)	11 (2–19)	14 (9–19)
Senses	24 (14–34)	33 (23–42)	51 (37–66)	54 (45–64)	44 (34–55)	50 (44–57)	33 (22–45)	34 (25–42)
Speech	15 (5–25)	12 (6–18)	13 (3–24)	32$\uparrow$** (23–40)	31 (2–61)	30 (22–38)	16 (1–31)	16 (11–21)
Social eating	17 (10–24)	24 (15–32)	33 (21–46)	52$\uparrow$* (43–62)	39 (26–52)	49$\uparrow$ (40–57)	20 (12–28)	27 (19–34)
Social contact	10 (1–19)	8 (4–12)	13 (4–22)	24$\uparrow$ (14–34)	10 (-3-23)	21$\uparrow$ (14–28)	2 (-2-5)	7 (3–10)
Sexuality	31 (17–46)	31 (19–43)	23 (6–40)	57$\uparrow$** (46–69)	37 (3–70)	45 (32–57)	17 (4–29)	23 (14–32)
**Single items**								
Problems with teeth	6 (0–13)	5 (0–10)	5 (-2-11)	17$\uparrow$* (10–23)	6 (-5-16)	13 (7–19)	10 (-2-21)	12 5–18
Problems mouth opening	6 (0–13)	16$\uparrow$ (7–25)	7 (0–15)	29$\uparrow$** (22–37)	6 (-5-16)	33$\uparrow$* (26–41)	14 (-4-32)	16 (10–22)
Dry mouth	31 (20–42)	45$\uparrow$ (34–57)	40 (28–53)	61$\uparrow$* (50–71)	61 (30–92)	66 (56–76)	57 (25–89)	64 (55–73)
Sticky saliva	35 (22–47)	44 (33–55)	36 (27–44)	71$\uparrow$** (61–80)	56 (28–83)	73$\uparrow$ (64–81)	33 (4–63)	51$\uparrow$ (40–62)
Coughing	15 (6–24)	15 (7–22)	29 (17–40)	32 (23–41)	39 (8–70)	42 (33–51)	14 (2–27)	22 (14–29)
Feeling ill	14 (7–22)	17 (8–27)	19 6–32	34 (25–44)	17 (-16-49)	38$\uparrow$ (28–47)	10 (-2-21)	9 (3–14)
Use of painkillers	38* (19–58)	78$\uparrow$* (62–95)	79 (56–101)	88 (77–99)	100 (100–100)	85 (74–96)	0 (0–0)	35$\uparrow$ 21–49
Nutritional support	27 (10–44)	42$\uparrow$ (22–61)	62 (35–88)	79$\uparrow$ (65–93)	83 (51–116)	65$\downarrow$ (50–80)	71 (38–105)	22$\downarrow$* (10–34)
Feeding tube	0.0 (0.0–0.0)	13$\uparrow$ (-1-26)	14 (-5-33)	29$\uparrow$ (14–45)	17 (-16-49)	48$\uparrow$ (32–63)	0 (0–0)	15$\uparrow$ (5–26)
Weight loss	19 (4–35)	38$\uparrow$ (18–57)	57 (30–84)	68$\uparrow$ (52–84)	67 (25–108)	63 (47–78)	14 (-12-40)	28$\uparrow$ (15–41)

Week 2: Data missing for 2 patients in the no/mild group and for 4 patients in the severe group (*n* = 57)

Week 4: Data missing for 3 patients in the no/mild group and for 6 patients in the severe group (*n* = 57)

Week 6: Data missing for 2 patient in the no/mild group and for 1 in the severe group. OMAS data is missing for 8 patients (*n* = 57)

3 months: Data missing for 2 patients in the severe group (*n* = 57)

High scores for functioning domains represents high level of functioning and high scores for symptom domains represent high levels of symptoms. The arrows show clinicicallly significant differences (> 10 points) between the patients with no/mild OM and those with severe oral mucositis. * = *p* < 0.05, ** = *p* < 0.01 (Mann Whitney U test)

### Weight loss and OM

From baseline to 2 weeks into treatment, 50% of the patients with no/mild OM (*n* = 13) had lost weight (mean weight loss 2.0 ± 1.1 kg), while 30% of the patients with severe OM (*n* = 9) experienced weight loss (mean weight loss 2.2 ± 1.6 kg). By 4 weeks into treatment, 62% of the patients with no/mild OM (*n* = 8) had lost a mean of 2.7 ± 2.4 kg, while 70% of the patients with severe OM (*n* = 26) had lost 3.2 ± 3.0 kg compared to baseline. At 6 weeks of treatment, 50% of the patients with no/mild OM (*n* = 3) had experienced a mean weight loss of 1.8 ± 1.1 kg, while 83% of the patients with severe OM (*n* = 34) had lost 4.2 ± 2.9 kg compared to baseline.

### Salivary secretion and OM

At two weeks into treatment the mean stimulated salivary secretion rate was 1.4 ml/min for the patients with mild OM (*n* = 25) and 1.3 ml/min for those with severe OM (*n* = 21). Further reduction in salivary secretion was observed at four weeks into treatment where the patients with mild OM (*n* = 13) showing a mean stimulated salivary secretion of 1.1 ml/min and the patients with severe OM (*n* = 31) 0.9 ml/min. By six weeks into treatment the patients with mild OM (*n* = 6) had a mean stimulated salivary secretion rate of 0.8 ± 0.5 ml/min whereas those with severe OM (*n* = 26) had 0.9 ± 0.7 ml/min. Approximately 50% of the patients in both the no/mild OM group (*n* = 3) and the severe OM group (*n* = 12) exhibited hyposalivation (< 0.7 ml/min) at that time-point.

## Discussion

To the best of our knowledge, this study represents the first analysis where both HR-QoL and clinician reported OM have been examined and HR-QoL has been compared between patients with no/mild OM and severe OM. This study found that patients with severe OM reported more problems with role functioning, social functioning, pain, fatigue, dry mouth, sticky saliva, problems with speaking, the need for nutritional support, the requirement of a feeding tube and issues with mouth opening particularly evident at weeks 4 and 6 during treatment (Tables 5 and 6) compared to those with no/mild OM. At three months post treatment the patients who had severe OM at week 6 during treatment reported more difficulties concerning sticky saliva, the need for a feeding tube and the use of painkillers, potentially contributing to the weight loss reported by these patients.

It is crucial to carefully monitor the development of OM and to encourage the patients to maintain good oral hygiene to reduce the bacterial load in the mouth, which could otherwise increase the risk of mucosal inflammation. Once OM has begun, bacteria can invade the ulcerations, exacerbating the spread of mucositis [35]. Studies have shown the significance of saliva secretion rate in the development and severity of oral mucositis [36]. A decrease in salivary flow may reduce the availability of antimicrobial and protective substances thus compromising the natural defense mechanisms in the oral cavity [37]. Therefore, it is essential to measure the patient’s salivary secretion before beginning oncological treatment to implement appropriate measures. These may include recommending frequent mouth rinsing especially after meals with solutions such as sodium chloride/bicarbonate [38], and to frequently lubricate the oral cavity with saliva substitutes. Other recommendations of prevention and treatment include photo-biomodulation as recommended by MASCC/ISOO practical guidelines. This form of therapy is however not part of the Swedish National Care Program for head and neck cancer 2022 [1] and was not considered for the patients included in the present study.

All patients included in the present study underwent a comprehensive oral/dental examination and received necessary dental treatment to prevent dental infections during oncologic treatment and to mitigate oral sequelae post treatment. This recommendation aligns with international guidelines for HNC patients [39, 40]. In Sweden, these patients receive dental examinations and treatment at a reduced cost, funded by the National Social Insurance. Consequently, patients do not incur expenses for visits to the dental clinic during oncological treatment or for the oncological treatment itself, facilitating access to oral care during treatment, likely benefiting HR-QoL.

Despite the fact that 65% of the patients having no comorbidities but their HNC cancer, had minimal medication intake and good oral health pretreatment, OM appeared as a side-effect of HNC treatment, and with a considerable proportion experiencing severe OM. Additionally, HR-QoL was negatively affected during oncological treatment with significant deteriorations of symptoms reported at week 4 of treatment and persisting at week 6, which is in congruence with other studies [14, 18, 19, 21, 22].

Dental status may affect symptoms and problems particularly regarding the HNC specific questionnaire (H&N35). Participants in this study exhibited good dental status with a high number of teeth (mean 27 and median 28), and good oral hygiene (low plaque scores 0.53 ± 0.60 and a low level of gingival inflammation 0.43 ± 0.51) (Table 2). This coupled with access to dental care during oncological treatment, may explain why the highest mean score for problems with teeth (such as pain when chewing) was only 14. Another Scandinavian study found a comparable mean score during and directly after completed oncologic treatment [14]. In two post RT studies involving patients in Taiwan [41] and India [42], higher scores for problems with teeth were reported, possibly attributable to poorer dental status pretreatment and limited access to dental care in those countries.

### HR-QoL and saliva

The reduced amount of saliva and its altered composition, as indicated by the patients’ experiencing sticky saliva severely affects quality of life and oral functioning. Saliva is crucial for bolus formation and thereby for the ability to swallow [37]. Saliva also aids in dissolving flavoring substances, potentially explaining why the patients reported sensory issues, especially with taste. Having a dry mouth and experiencing swallowing difficulties combined with altered taste makes social eating unpleasant, contributing to patient discomfort. A study by Epstein et al. [43] found that HNC patients undergoing oncological treatment experienced changes in umami taste (amino acids) and lipids (linoleic acid). Oral pain and appetite loss further affect the willingness to eat leading to fatigue and weight loss. Therefore, it is likely that most of the problems and symptoms reported by patients during oncological treatment stem from the marked reduction in salivary secretion rate in combination with other side-effects of the oncological treatment.

### HR-QoL three months post treatment

Three months post treatment, the scores on the functioning scales was comparable with baseline, which is in congruence with previous studies [14, 21, 44]. However, the patients in this study still reported problems with appetite loss, senses, social eating, dry mouth and sticky saliva three months post treatment, aligning with findings from other studies [14, 15, 17, 19, 21]. The reported improvement in HR-QoL outcomes by the patients could be attributed to psychological adaptation to their new circumstances, enabling them to better cope, a phenomenon known as response shift [45]. This could lead to recall bias, although no tests were conducted to detect this in the present study.

Patients with severe OM at week 6 of oncological treatment reported significant problems with appetite loss, sticky saliva, use of painkillers, need for a feeding tube and weight loss persisted three months post treatment, thus highlighting the importance of continued follow up and support after completing cancer treatment.

The parotid- and submandibular glands are organs at risk for damage from RT [46, 47]. As mentioned earlier a salivary gland sparing technique with IMRT and VMAT was used. In the present study, the mean salivary secretion rate was 0.7 ml/min 3 months post treatment and 57% had hyposalivation, likely contributing to the patients’ persistent problems with dry mouth, sticky saliva, and appetite loss. In a qualitative study one informant describes the sensation of eating crisp akin to swallowing nails [48]. Concerns about weight loss, insufficient nutrient intake, and fear of choking on food getting stuck in the throat were also described by the informants [48].

Difficulties with eating may lead to a diet rich in soft foods, often high in carbohydrates. A low salivary secretion rate prolongs periods of low pH after meals due to reduced oral clearance and bacterial metabolism of carbohydrates potentially increasing the risk of caries and dental erosion [49]. In many cases, the salivary secretion rate does not improve over time. In our previous study, 50% of the patients still had hyposalivation two years post treatment and reported problems with dry mouth [50].

The low salivary secretion rate likely contributed to persistent appetite loss and weight loss found in the present study. At six weeks into treatment 83% of the patients, (who also suffered from severe OM), had lost an average of 4.2 ± 2.9 kg compared to baseline. At three months post treatment the mean weight for all patients was significantly lower regardless of OM severity, 79 ± 13 kg compared to 85 ± 14 kg at baseline. Long-term nutritional follow-up by a dietician after completion of cancer treatment is important to reduce the risk of malnutrition and unintentional weight loss as recommended by the Confederation of Regional Cancer Centers in Sweden; National care program for head and neck cancer (2022) [1] and supported by previous studies [51, 52].

It is important for both dental- and health care professionals to monitor patients during and after completing oncologic treatment to detect symptoms and problems that may impede rehabilitation, temporarily or permanently, and to provide advice and recommendations to aid the patients. Dry mouth and low salivary secretion rate are common long-term side effects after HNC treatment. It has been reported by patients that their experience of living with a dry mouth is not taken seriously by the healthcare system, and that living with a dry mouth is seen as a “small price to pay” by health care personnel considering the patients cancer cure [53].

### Methodological considerations

The reason for having the inclusion criteria ≥ 16 teeth was because there are few edentulous patients in Sweden. Another reason was that having few teeth may “skew” the HR-QoL domains regarding ability to eat towards lack of teeth instead of showing problems related to pain, lack of saliva or other problems and symptoms related to cancer treatment. A large variation in number of teeth may also influence the prevalence of OM as it is known that inadequate oral hygiene and pathogenic microorganisms plays a role in the severity and spread of OM. It has been shown that there is a strong correlation between the Oral mucositis assessment scale (OMAS) score recorded by health care professionals and the symptoms reported by patients [24]. The most used scales for assessing the severity of OM include OMAS, the World Health Organization Oral Toxicity Scale (WHO-OTS), Radiation Therapy Oncology Group instrument (RTOG) and Common Terminology Criteria for Adverse Events (CTCAE). This study opted to utilize OMAS due to its comprehensive description of the extent and severity of OM, including a sub analysis of mucosal damage measuring erythema and ulcerations across anatomic sites. To our current knowledge, there are no guidelines regarding scores for mild and severe OM. Therefore, we assigned ulceration scores of 0–1 as no/mild OM ulceration scores ≥ 2 as severe OM. Assessment of the unstimulated salivary secretion rate was deemed as too exhausting for the patients in regard to all clinical parameters that was collected and was as such not considered for the study. It is also likely that the unstimulated secretion rate would be unmeasurable or very low as shown in our previous study of HNC patients post treatment [54].

The strengths of the present study lie in the assessment that dental status, oral hygiene level, salivary secretion rate and weight were registered and followed at various time-points during treatment.

## Future perspectives

Despite favorable oral and dental status, OM remained a prevalent issue for patients in this study during oncological treatment. This ongoing study is evaluating the effects of an intensified oral care program for HNC patients to alleviate OM. Studies using qualitative methodology are suggested to provide a better understanding of patient’s strategies to deal with persistent problems after oncologic treatment aiming at improving care and quality of life for this population. How patient’s oral hygiene behaviours may influence the prevalence of plaque and gingivitis and the impact on the severity and duration of oral mucositis is another area for further research.

## Conclusion

In conclusion, the study demonstrates that the impact of OM on HR-QoL extends beyond local oral complications and affecting global QoL. Patients’ experienced exacerbated symptoms and problems weeks 4 and 6 of oncological treatment, especially among those with severe OM stressing the importance of clinically monitoring the patients to reduce and alleviate their symptoms. Persistent problems three months post treatment are likely associated with the reduced salivary secretion rate indicating that patients should be monitored also after completed oncological treatment.

## Data Availability

The datasets used and/or analysed during the current study are available from the corresponding author upon reasonable request.
